# The Stability of Complement-Mediated Bactericidal Activity in Human Serum against *Salmonella*

**DOI:** 10.1371/journal.pone.0049147

**Published:** 2012-11-08

**Authors:** Colette M. O’Shaughnessy, Adam F. Cunningham, Calman A. MacLennan

**Affiliations:** 1 Medical Research Council Centre for Immune Regulation and Clinical Immunology Service, Institute of Biomedical Research, School of Immunity and Infection, College of Medicine and Dental Sciences, University of Birmingham, Birmingham, United Kingdom; 2 Novartis Vaccines Institute for Global Health, Siena, Italy; University of Padova, Medical School, Italy

## Abstract

The complement cascade includes heat-labile proteins and care is required when handling serum in order to preserve its functional integrity. We have previously used a whole human serum bactericidal assay to show that antibody and an intact complement system are required in blood for killing of invasive isolates of *Salmonella*. The aim of the present study was to evaluate the conditions under which human serum can be stored and manipulated while maintaining complement integrity. Serum bactericidal activity against *Salmonella* was maintained for a minimum of 35 days when stored at 4°C, eight days at 22°C and 54 hours at 37°C. Up to three freeze-thaw cycles had no effect on the persistence of bactericidal activity and hemolytic complement assays confirmed no effect on complement function. Delay in the separation of serum for up to four days from clotted blood stored at 22°C did not affect bactericidal activity. Dilution of serum resulted in an increased rate of loss of bactericidal activity and so serum should be stored undiluted. These findings indicate that the current guidelines concerning manipulation and storage of human serum to preserve complement integrity and function leave a large margin for safety with regards to bactericidal activity against *Salmonella*. The study provides a scheme for determining the requirements for serum handling in relation to functional activity of complement in other systems.

## Introduction

Complement comprises a set of plasma proteins that facilitate the killing of pathogens by antibody [1], [2]. This involves a number of mechanisms including cell-independent bactericidal activity with formation of membrane attack complex, and opsonization for uptake and killing by phagocytic cells. We have previously studied the ability of whole human serum from Africans to effect cell-independent and facilitate cell-mediated killing of African invasive isolates of nontyphoidal *Salmonella*. We have found that these mechanisms of bacterial killing require both antibody and complement [3], [4].

It has long been known that complement activity is heat-labile [5], [6] and is destroyed by exposure to 56°C for 30 minutes, while antibody is relatively resistant to such treatment [7]. Therefore, careful processing and manipulation of blood and serum for use in functional complement assays is required to avoid false-negative results [8]. Thus it is advocated that serum is promptly separated from clotted blood and stored at −80°C in aliquots to avoid freeze-thaw cycles. It is also normal practice for serum aliquots to only be used on the day they are thawed, and that experiments involving complement are carried out on ice or under refrigerated conditions at 4°C.

We have been able to uncover only limited objective data about the stability of the human complement components required to induce cell free and phagocyte-mediated lysis of bacteria in vitro. Hemolytic assays are normally used for such investigations of complement stability [6] and these normally focus on animal serum, in particularly guinea pig serum [6]. There are several studies showing functional differences in complement between species including humans [9].

We conducted the current study to determine acceptable conditions for manipulation of human serum that would not be detrimental to its ability to kill *Salmonella* in our cell-free bactericidal assays. In this study, we explored the longevity of bactericidal activity at different temperatures, effect of freeze-thaw cycles and effect of delayed separation of serum from blood. We were able to determine the conditions for preservation of serum complement activity for use in our assay system. The study also serves to provide a scheme for assessing serum handling in order to maintain complement function for other in vitro functional assay systems.

**Figure 1:**
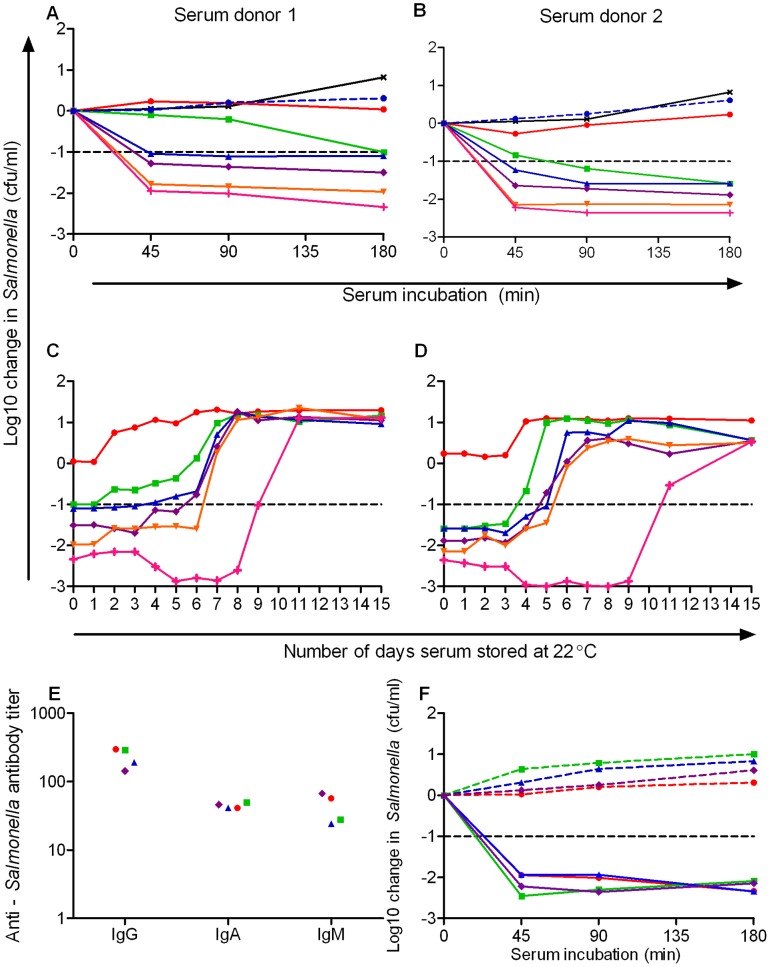
Percentage of human serum stored at 22°C required to kill *S*. Typhimurium D23580 and anti-*S*. Typhimurium antibody levels in study sera. Killing of bacteria by different percentages of freshly thawed serum from *A*. subject 1 and *B*. subject 2 at 45, 90 and 180 minutes in serum bactericidal assay using 10^6^ cfu/ml *S*. Typhimurium D23580∶10% (red circles), 20% (green squares), 30% (blue triangles), 40% (purple diamonds) and 50% (orange inverted triangles) serum diluted with PBS, 100% PBS (black crosses), 100% fresh serum (pink vertical crosses) and 100% heat-inactivated serum (blue circle and dashed lines). Initial concentration of bacteria 10^6^ cfu/ml with numbers of viable bacteria plotted against duration of assay. Killing of bacteria by different percentages of serum from *C*. subject 1 and *D*. subject 2 at 180 minutes following storage at 22°C. Change in viable count (cfu/ml) plotted against duration of time serum stored at 22°C. *E*. Levels of anti-*S*. Typhimurium D23580 IgG, IgA and IgM antibodies in human sera used in study as assessed by flow cytometry. *F*. Killing of bacteria by fresh serum (solid lines) and heat-inactivated serum (dashed lines) from subject 1 (red circles), 2 (green squares), 3 (blue triangles) and 4 (purple diamonds) at 45, 90 and 180 minutes.

**Figure 2:**
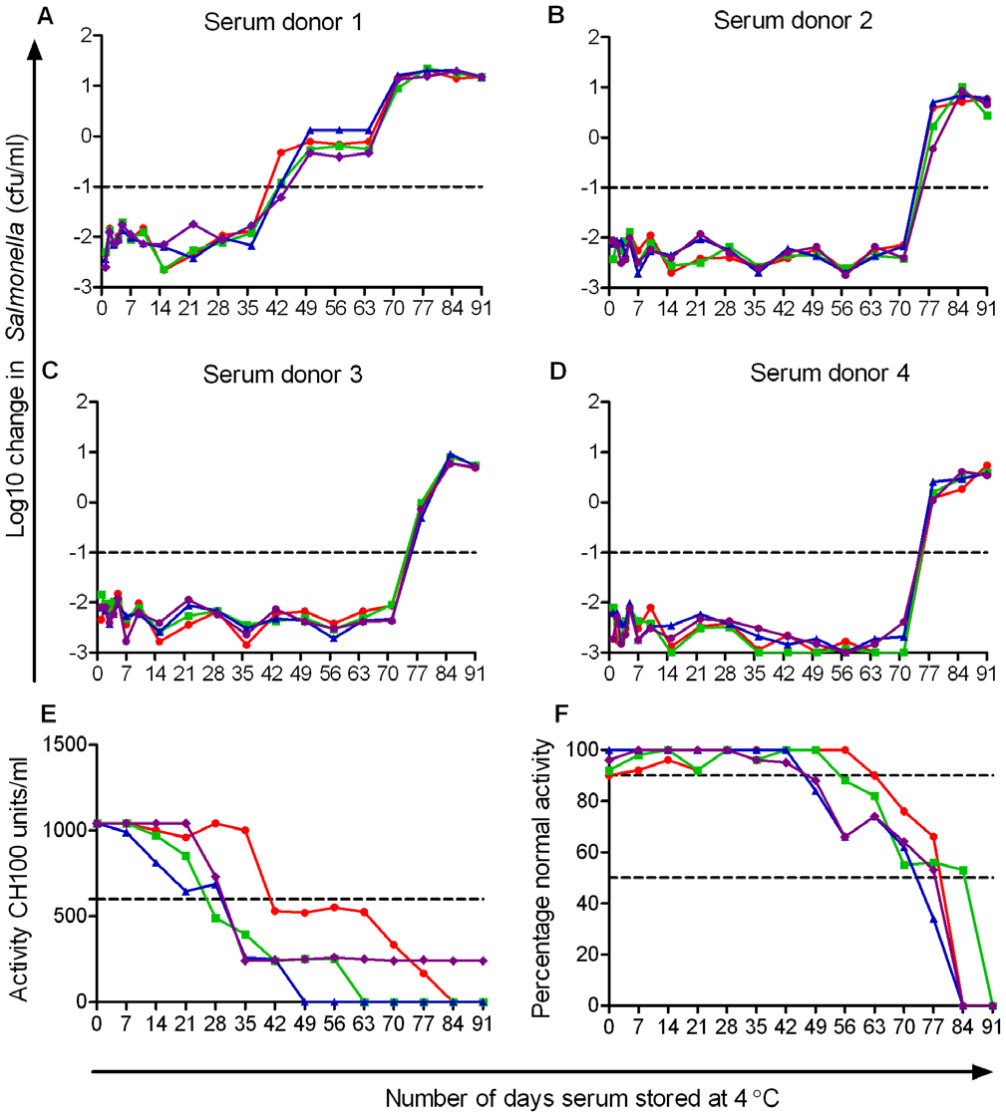
Effect of duration of storage of human serum at 4°C and freeze-thaw cycles on its ability to kill *S*. Typhimurium D23580. Serum from four healthy adults (subjects 1–4 corresponding with panels *A*.–*D*.) following one (green squares), two (blue triangles), three (purple diamonds) or no (red circles) freeze-thaw cycles was maintained at 4°C and examined at intervals for ability to kill in the serum bactericidal assay. Normal killing designated as a reduction of 1.0 log10 cfu/ml viable bacteria. Numbers of viable *Salmonellae* after 180 minutes in the assay are plotted against number of days serum kept at 4°C. Negative values show a decrease in viable bacteria compared with initial concentration. Classical (*E*.) and alternative (*F*.) pathway hemolytic complement activity in sera from the four subjects (1– red circles, 2– green squares, 3– blue triangles, 4– purple diamonds) stored at 4°C.

## Materials and Methods

### Ethical Approval

Ethical approval for the use of serum samples in this study was granted by the Life and Health Sciences Ethical Review Committee of the University of Birmingham. Informed written consent was obtained from all participants.

### Serum

Blood was venesected from four healthy adults (two male, two female) and left to clot at 4°C for 8 hours or at 22°C for from zero to four days in the delayed serum-separation experiment. Serum was separated by centrifugation at 4°C and frozen in aliquots at −80°C in sterile 8 ml polypropylene tubes (Sarstedt) or left at 4°C or 22°C (in the delayed serum-separation experiment). When required, frozen aliquots of serum were thawed on the bench at 22°C. Samples undergoing freeze-thaw cycles were immediately refrozen at −80°C after reaching 22°C, and this was repeated daily for up to three sequential days. Heat-inactivation of complement was performed, when needed, by incubating serum in a water bath at 56°C for 30 minutes.

**Figure 3:**
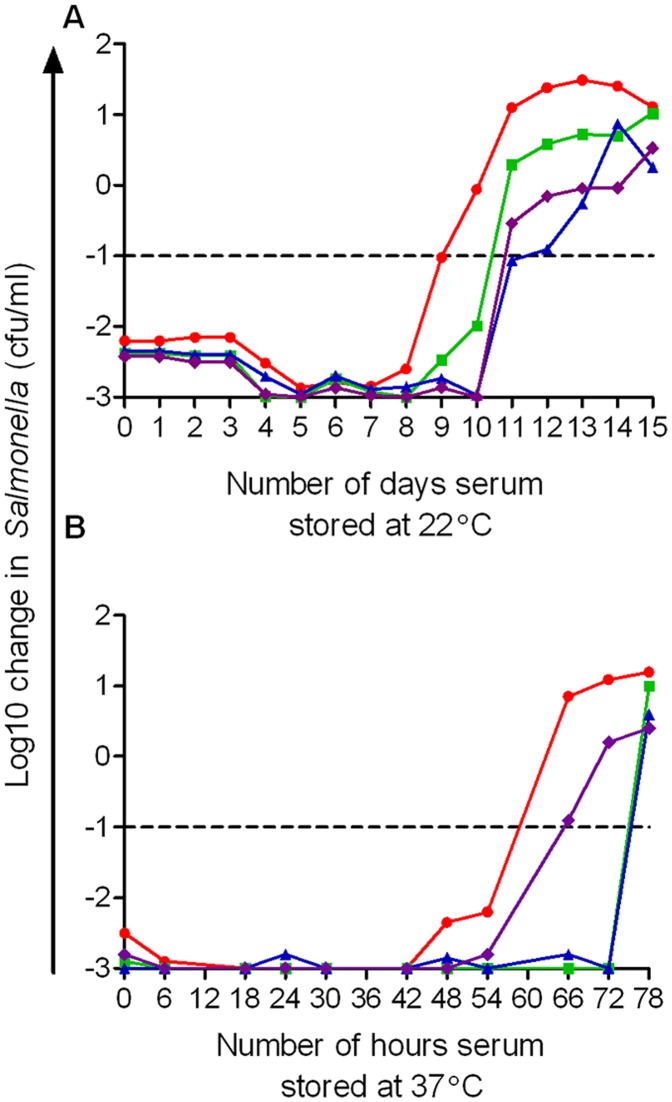
Effect of duration of storage of human serum at 22°C and 37°C on ability to kill *S*. Typhimurium D23580. Serum from the same four adults as in Fig. 1 (1– red circles, 2– green squares, 3– blue triangles, 4– purple diamonds) was thawed and maintained at 22°C (*A*.) or 37°C (*B*.) with serum bactericidal assays performed daily (for samples at 22°C) or six- to twelve-hourly (for samples at 37°C) using 10^6^ cfu/ml. Viable bacteria at 180 minutes in each serum bactericidal assay are plotted against duration of serum storage.

**Figure 4:**
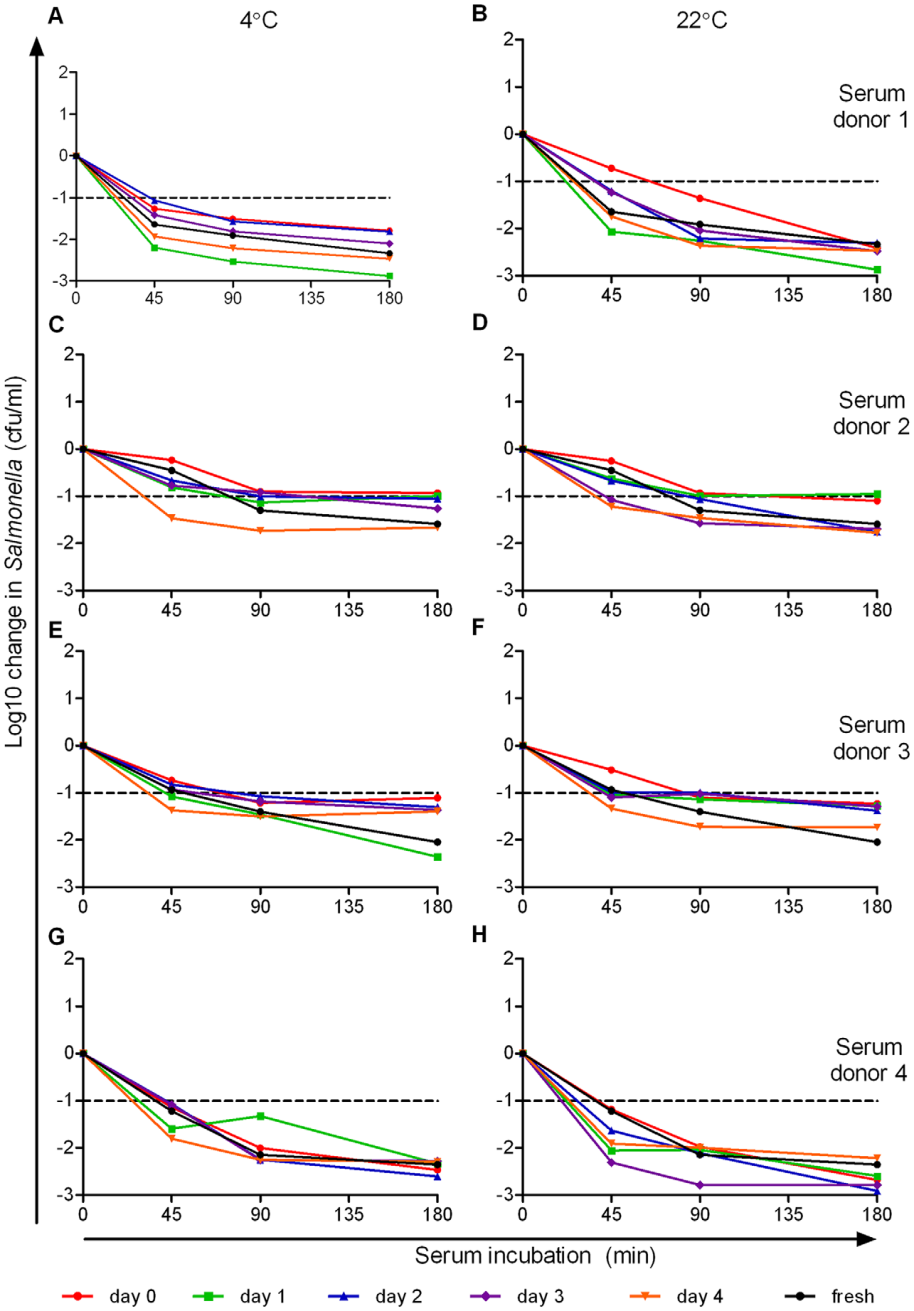
Effect of delayed separation of serum from clotted human blood on ability to kill *S*. Typhimurium D23580. Blood from four healthy adults (subjects 1–4 corresponding with panels *A*.–*B*., *C*.–*D*., *E*.–*F*. and *G*.–*H*. respectively) was kept at 4°C (*A*., *C*., *E*., *G*.) or 22°C (*B*., *D*., *F*., *H*.) post-venesection and serum separated the same day (red circles) or after one (green squares), two (blue triangles), three (purple diamonds ), or four (orange inverted triangles) days after venesection. Separated sera were then maintained at 4°C without freezing for a total of four days post venesection before being used in the serum bactericidal assays and compared with fresh serum from each donor (black circles). Changes in cfu/ml are plotted against the duration of assay.

### Salmonella

*Salmonella enterica* serovar Typhimurium D23580, a well-characterized invasive nontyphoidal *Salmonella* isolate from Malawi [3], [10], was used throughout the study. D23580 is sensitive to antibody-dependent complement-mediated killing, undergoing a one to three log10 reduction in bacterial numbers within three hours of exposure to serum from healthy adults at 37°C [3].

### Serum Bactericidal Assays

Serum bactericidal assays against *Salmonella* were as described previously [3]. *S*. Typhimurium D23580 was grown at 37°C to log-phase from an overnight culture in LB medium for two hours and washed twice in PBS. 10 µl of washed bacteria resuspended in PBS, without additional Mg^2+^ or Ca^2+^, was added to 90 µl of serum in a microfuge tube to give a final concentration of 10^6^ cfu/ml in 90% serum, and incubated on a rocker plate at 37°C and 20 rpm. Viable numbers of *Salmonellae* were determined for the washed bacterial inoculum and at 45, 90 and 180 minutes exposure to serum by serial dilution in PBS followed by overnight growth at 37°C on LB agar. Killing of *Salmonellae* was calculated by subtracting the concentration of viable *Salmonellae* at the start of the assay from the concentration of viable bacteria at each time point. Serum bactericidal assays were performed with heat-inactivated serum as a negative control. When required, for inhibition of alternative pathway activity, polyclonal antibodies to Factor B (ab8840, Abcam, Cambridge, UK) were added to the assay at a final concentration of 0.92 mg/ml.

**Figure 5:**
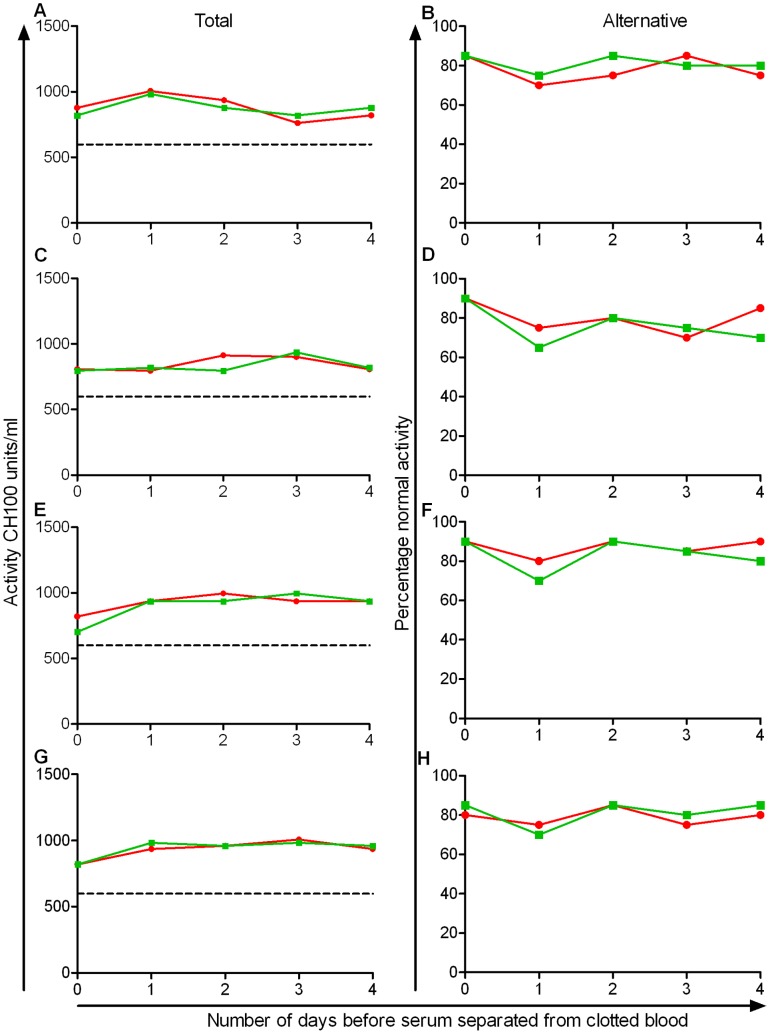
Effect of delayed separation of serum from clotted human blood on classical and alternative pathway hemolytic complement activity. Classical (*A., C., E., G.)* and alternative (*B., D., F., H.*) pathway hemolytic complement activity in sera from four subjects (corresponding with panels *A–B, C–D, E–F, G–H*) separated from clotted blood on the day of venesected (day 0) or stored at 4°C (red circles) or 22°C (green squares) and separated 1–4 days post-venesection. Once separated, all samples were maintained at −80°C until tested in the radial immunodiffusion assays.

### Anti-*Salmonella* Antibody Assays

Anti-*S*. Typhimurium D23580 IgG, IgA and IgM levels were assessed by flow cytometry as previously described [3].

### Complement Hemolytic Assays

Classical and alternative pathway hemolytic complement assays were performed by radial immunodiffusion (Binding Site, UK) according to the manufacturer’s instructions.

### Statistics

The ANOVA test was used to compare groups of data within the study using Prism version 5.02 (GraphPad Software, California).

**Figure 6:**
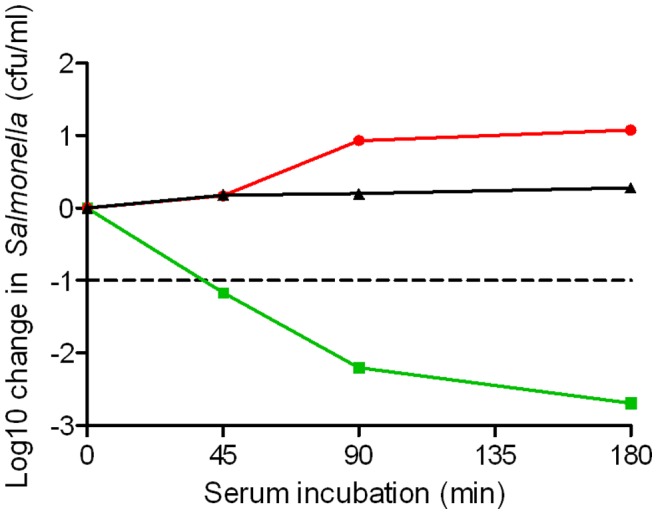
Effect of presence of Factor B antibody on ability of human serum to kill *S.* Typhimurium D23580. Killing of bacteria by 90% fresh serum from subject 1 and 10% polyclonal anti-Factor B antibody (final concentration 0.92 mg/ml) (red circles), compared with 90% fresh serum from subject 1 and 10% PBS (green squares) and 100% heat-inactivated serum (black triangles) at 45, 90 and 180 minutes in the serum bactericidal assay using 10^6^ cfu/ml *S*. Typhimurium D23580.

## Results

### The Percentage of Human Serum Required to Kill *Salmonella* Increases Incrementally with Time

The ability of fresh adult human serum to kill *Salmonella* has previously been shown to depend on specific IgG or IgM antibody activating the classical complement pathway and the consequent deposition of membrane attack complex on the bacterial surface [3]. Serum samples from two healthy adults (subjects 1 and 2) were studied for their ability to kill *S*. Typhimurium D23580 when diluted in PBS. We confirmed the finding that, when first thawed, serum concentrations of 20% or above are required to achieve a 1.0 log10 cfu/ml kill of *Salmonella* (designated ‘normal’ killing) at 180 minutes in the serum bactericidal assay (Fig. 1*A–B*) [3]. Bacterial viability in 10% serum is similar to that of bacteria cultured in complement-depleted serum.

When subsequently kept at 22°C, the ability of the diluted sera to effect a normal kill decreased more rapidly than with whole serum. The most dilute sera lost the ability to kill first: 20% sera was unable to effect normal killing after one to three days, 30% serum after three to four days, 40% serum after four to five days and 50% after five to six days (Fig. 1*C–D*).

### Human Sera used in the Study Contained *Salmonella*-specific Antibody

In order to ascertain that all sera used in the study contained antibody directed against *Salmonella*, we assessed the presence of anti-*Salmonella* antibody targeting *S*. Typhimurium D23580 by flow cytometry. Sera from all four healthy adult subjects contained IgG, IgA and IgM classes of antibody capable of binding D23580 (Fig. 1*E*), indicating that these sera could activate complement deposition on D23580 via the classical complement pathway [3]. To ensure that reductions in viable bacterial numbers in the serum bactericidal assay were complement-dependent and did not result from antibody alone, serum bactericidal assays were performed following heat-inactivation of each serum. Bacterial numbers increased relative to the starting concentration at each time point in each serum (Fig. 1*F*).

### Serum Bactericidal Activity Against *Salmonella* is Retained for at least 35 days at 4°C

After these preliminary studies, further analysis of the decline in bactericidal activity on storage were carried out using freshly-frozen aliquots of serum from the four subjects and assessing the bactericidal capacity of undiluted serum. Aliquots were subjected to one, two, three or no freeze-thaw cycles and then maintained at 4°C with the serum bactericidal assays against *S*. Typhimurium D23580, being repeated daily for the first week and then weekly. During the first five weeks of the experiment (up to day 35), each serum killed D23580 by approximately 2.0 log10 cfu/ml (Fig. 2*A–D*). Thereafter, serum from subject 1 was unable to effect a 1.0 log10 cfu/ml kill (Fig. 2*A*), while serum from the remaining three subjects maintained killing for 70 days (Fig. 1*B–D*) The serum from subject 1 had no effect on the ability of other sera to kill *Salmonellae* when combined with other sera in different proportions in the serum bactericidal assay. There was no detectable difference in the ability of serum that had undergone zero, one, two or three freeze-thaw cycles to kill *Salmonella* D23580 (Fig. 2*A–D*).

A gradual reduction in classical pathway hemolytic complement activity was observed with serum from each subject. Each serum had over 1000 CH100 units/ml on day 0, less than 600 CH100 units/ml by day 28 to 42 and serum from 3 of the subjects had no detectable activity on day 49, 63 or 84 (Fig. 2*E*). One serum (from donor 4) still had detectable classical pathway activity at the end of the experiment (day 91). Alternative pathway hemolytic complement activity was maintained at over 90% normal activity for 42 days in all sera, fell to less than 50% by day 77 and no activity was detectable at 84 or 91 days (Fig. 2*F*).

### Serum has Reduced Longevity of Bactericidal Activity Against *Salmonella* when Stored at 22°C and 37°C

To investigate the effect of increasing the temperature at which serum is stored on its bactericidal activity against *S*. Typhimurium, the above experiment was repeated with serum from the same four subjects maintained at either 22°C (Fig. 3*A*) or 37°C (Fig. 3*B*) post-thawing. Sera kept at 22°C could effect a 2.0 to 3.0 log10 kill for 8 to 10 days after which bactericidal activity fell to less than 1.0 log10 cfu/ml. Sera kept at 37°C were assessed six- to twelve-hourly for bactericidal activity against D23580. At this temperature, bactericidal activity fell more rapidly than at 22°C and was less than 1.0 log10 cfu/ml after 54 hours for two sera and after 72 hours for the other two sera.

### Delayed Separation of Serum from Clotted Human Blood does not Reduce Ability to Kill *Salmonella*

Delayed separation of serum from blood often occurs due to delays in transportation of blood samples from clinic to laboratory. To investigate the effect of up to four days delay in separation of serum from blood on *Salmonella* killing, we performed the serum bactericidal assay using blood kept at either 4°C (Fig. 4*A, C, E, G*) or 22°C (Fig. 4*B, D, F, H*) post-venesection. Blood was separated either on the day of venesection or after one to four days, and, following separation, sera were stored at 4°C. Assays were performed on all samples a total of four days post-venesection. Compared with fresh sera, there was no significant difference in the bactericidal activity of the serum after 1, 2, 3 or 4 days’ delay in separation from whole blood at any of the three assay time points (45, 90 and 180 minutes), regardless of whether the blood had been stored at 4°C (ANOVA, *p* = 0.09 to 0.74) or 22°C (*p* = 0.19 to 0.99).

Loss of complement activity following four days of delayed separation of serum from blood could occur to a degree that would not be detectable by our *Salmonella* killing assay. To test this, we took further samples of blood from the four donors and separated and froze serum on the day of venesection, and after 1, 2, 3 or 4 days at 4°C and 22°C for functional complement pathway assays. There was no significant difference in classical or alternative pathway hemolytic complement activity with serum that had been separated from blood after four days at 4°C or 22°C, compared with serum separated and frozen on the day of venesection (Fig. 5).

### Blocking of Alternative Pathway Activity with Anti-properdin Antibodies Inhibits Serum Killing of *Salmonella*

In order to test whether killing of *S*. Typhimurium D23580 was dependent on the alternative pathway of complement, we performed the serum bactericidal assay with anti-properdin antibodies added to fresh serum from subject 1 at a final concentration of 0.92 mg/ml in order to block the alternative pathway. Killing of *Salmonella* was fully inhibited with net bacterial growth at all time points in the assay (Fig. 6).

## Discussion

Despite the importance given to careful handling of serum in order to preserve complement activity, the available scientific literature describing experimental studies on this activity dates from the first half of the 20^th^ century. Much of the data described in this literature relates to studies of animal, rather than human complement, and uses hemolytic complement assays that may not reflect the bactericidal assay against *Salmonella* or bacteria in general, which usually depend on the combined action of antibody and complement.

A common assumption is that frozen aliquots of serum should be used on the day of thawing when required for bactericidal assays. Our findings indicate that this is not the case, since thawed serum stored at 4°C effects normal levels of killing of *Salmonella* for 35 days. For three of the four sera, killing was still obtained after 70 days at 4°C. We have previously shown that classical pathway of complement is required for bactericidal activity against invasive African isolates of *Salmonella* [3]. These current findings are consistent with only a small level of classical pathway activity being required for this activity, a level that can be undetectable by hemolytic radial immunodiffusion assay. Another assumption is that serum must be separated from blood immediately after clotting and stored at −80°C until use in order to retain complement function. We found no loss of ability to kill *Salmonella* in our serum bactericidal assay using blood that had been left for four days at room temperature prior to serum separation and use. It is possible that some loss of complement activity, undetectable by our serum bactericidal assay, which standardly uses undiluted serum, may have occurred during this time. However, the lack of change in functional complement activity assessed by the hemolytic assays indicates that this was not the case.

Mechanistically, these data are compatible with the hypothesis that once the classical pathway has initiated C3b deposition on *Salmonella*, this is sufficient for the deployment of the alternative pathway which then becomes the critical determinant of bactericidal activity. When we performed the bactericidal assay with anti-properdin antibodies to block the alternative pathway, killing was fully inhibited confirming the critical role of alternative pathway in serum killing of *Salmonella*. It is surprising that serum from subject 1 had only half the duration of bactericidal activity of the other three sera when stored at 4°C. Our data do not provide an explanation for this, since longevity of classical and alternative pathway hemolytic complement activity for this serum was no less than that for the other three sera, and there was no evidence for the blocking activity which we have previously described in serum from some HIV-infected African adults [11]. The difference in longevity of bactericidal activity between this serum and the others was less apparent at 22°C and 37°C.

Perhaps the most surprising finding of the study was the lack of any discernible effect on bactericidal activity of up to three freeze-thaw cycles, given the accepted view that freeze-thaw cycles are to be avoided in the context of preservation of complement function [8]. Consistent with this finding, a report from 1927 found that guinea pig serum could be subjected to twelve freeze-thaw cycles without an appreciable decrease of hemolytic activity [12]. As has previously been described using hemolytic assays [5], [6], we found that the longevity of bactericidal activity of human serum against *Salmonella* was reduced with increased storage temperature post-thawing. The practical implication of this is that if thawed human serum is to be used over an extended number of days, it is important that it is kept refrigerated.

When considering the significance of the findings of the current study, it is important to consider that much of the non-variable blood and serum handling involved was conducted under optimal conditions. Different findings could be expected if, for example, frozen serum had been stored at temperatures higher than −80°C, or if during freeze-thaw cycles, serum had been left thawed for extended periods of time before refreezing. We have allowed blood to clot at both room temperature and 4°C, as described in the present study, and have found no difference in integrity of complement activity with either protocol. Blood clots more rapidly at room temperature and should be separated and frozen sooner than blood stored at 4°C. As expected, diluted serum loses bactericidal activity more rapidly than undiluted serum, so serum to be used for serum bactericidal assays and other functional complement assays should be stored undiluted. It is important to remember that human complement does not necessarily behave the same as animal complement in bactericidal assays [9]. Nevertheless, the findings of the study indicate that the current guidelines concerning manipulation and storage of serum to preserve complement integrity and function leave a large margin for safety.

It possible that serum from some individuals may perform less well that than that of others due, for example, to polymorphisms of the alleles encoding C4. Furthermore, serum from individuals with complement-consuming conditions such as autoimmunity or hepatitis C infection would be unsuitable for use. The current study only tested serum from four individuals which was due to the large number of assays required to determine the conditions for serum manipulation. Ideally, serum for each new donor should be tested in an established assay system prior to using it routinely.

Serum bactericidal assays, particularly against meningococcus, often use animal complement as a source of exogenous complement usually in the form of baby rabbit serum. While this can facilitate standardization, it has led to problems with assay sensitivity and specificity leading some to propose the use of human complement instead [13]. The current study supports the use of human serum as a source of complement for bactericidal assays by demonstrating the robust characteristics of human complement. Moreover, in serum bactericidal assays such as the one described to examine killing of *Salmonella*, the subject’s own endogenous complement is used allowing assessment of the combined activity of the subject’s antibody and complement in the bactericidal process. This could provide a more accurate indication of the complement-mediated bactericidal activity that each individual has against a pathogen.

While the current study was focused primarily on the preservation of complement activity in relation to bactericidal activity of human serum against *Salmonella*, it is important to establish the conditions for serum handling to each particular in vitro assay system and complement source. This study provides a model for the testing of conditions of serum manipulation that can be applied to other assay systems and other sources of complement.
